# Effects of Laser Welding and Post-Weld Heat Treatment on Microstructure and Mechanical Properties of Aged Ti55531 Alloy

**DOI:** 10.3390/ma11101907

**Published:** 2018-10-08

**Authors:** Lin-Jie Zhang, Jun-Yu Pei, Jian Long, Miao-Xia Xie, Xiang-Tao Shang, Jun Wu

**Affiliations:** 1 State Key Laboratory of Mechanical Behavior for Materials, Xi’an JiaoTong University, Xi’an 710049, China; zhanglinjie@mail.xjtu.edu.cn (L.-J.Z.); longjian528@stu.xjtu.edu.cn (J.L.); 2 School of Mechanical and Electrical Engineering, Xi’an University of Architecture and Technology, Xi’an 710055, China; Xiemiaoxia@xauat.edu.cn (M.-X.X.); shang_x_t@163.com (X.-T.S.); 3 Xi’an Aerospace Power Machine Factory Co., Ltd., Xi’an 710025, China; wujunyy@126.com

**Keywords:** Ti-5Al-5Mo-5V-3Cr-1Zr, laser welding, post-weld heat treatment, microstructure, mechanical properties

## Abstract

As one of the relatively new titanium (Ti) alloys in the engineering field, β-Ti alloy–Ti55531 has attracted a great deal of attention due to its excellent mechanical properties, while a few research papers on weldability and the post-weld heat treatment (PWHT) process of Ti55531 have been reported. Based on an orthogonal experiment design, the parameters of laser beam welding (LBW) of Ti55531 alloy with a thickness of 2 mm were optimized. Moreover, the influences of welding parameters and PWHT on the microstructures and performance of the laser-welded joint of Ti55531 were analyzed. The results showed that, for microstructures in different zones of as-welded joints of Ti55531: three forms of α phases (i.e., equiaxial α_p_ phase, lamellar α_S_ phase, and α_GB_ phase at grain boundaries) were observed in base metal (BM); in the heat affected zone (HAZ), part of lamellar α_S_ phase had dissolved while equiaxial α_p_ phase had grown; the fusion zone (FZ) mainly consisted of β phase, which presented as coarse columnar crystals. After the PWHT process, the microstructures of the welded joint were changed: in the BM zone, α phase at grain boundary disappeared and lamellar α phase decreased; in the HAZ, the edge of α_p_ phase obviously dissolved; in the FZ, plenty of compact needle-like α phases were observed. The tensile strength of the as-welded joint was about 940 MPa and then increased to 1161 MPa after PWHT, which were 78.4% and 96.8% of that of the original BM respectively. The fracture position transformed from the interface between the FZ and HAZ to the BM during tensile tests after PWHT.

## 1. Introduction

Some important structural parts, such as airframes and engines, need to achieve light weight by applying materials with high strength and low density [1,2]. As a novel metastable β-Ti alloy, Ti55531 was co-developed by VSMPO Company in Russia and Airbus S.A.S and applied to the Airbus A380. The nominal component of Ti55531 alloy is Ti-5Al-5Mo-5V-3Cr-1Zr. This type of Ti alloy without an Fe element not only shows the advantages of traditional high-strength and high-toughness Ti alloys but also avoids the drawbacks of the majority of traditional Ti alloys. For example, Ti55531 alloy exhibits various advantages including less sensitivity to segregation, good hardenability, excellent strength and high fracture toughness, and thus it is extremely applicable for manufacturing parts bearing huge stress, such as structural parts, landing gears, wings of airplanes, and engine pylon [3]. In the past several years, the regulation of microstructures and performance of Ti55531 alloy have been of wide concern to researchers [4,5,6,7,8,9,10]. However, the research mainly focused on base metal (BM) itself while few studies on microstructures and the performance of welded joints of Ti55531 alloy were reported. In order to reduce the weight of aircrafts, replacing riveting with welding will become a trend in the future and therefore it is necessary to investigate the weldability of Ti55531, the evolution of microstructures and performance of the welded joint [11]. 

The weldability of metastable β-Ti alloy has been a topic concerning researchers for several decades. Becker and Baeslack et al. (1993) investigated gas tungsten arc welding (GTAW) of metastable β-Ti alloy Beta-21S (Ti-15 wt % Mo-2.7 wt % Nb-3 wt % A1-0.2 wt % Si) and validated the favorable weldability of the type of Beta-21S [12]. By comparing laser beam welding (LBW), electron beam welding (EBW) and GTAW of metastable β-Ti alloy Ti5553, Pasang et al. found that coarse columnar grains appeared in the fusion zone (FZ) of welded joints whichever welding method was applied and the FZ in as-welded joints was subjected to significant softening. They found that owing to LBW and EBW exhibiting a lower heat input, and thus a narrower FZ, these two methods can yield welded joints with a superior performance to that obtained through GTAW. Therefore, at present, the welding methods frequently used for metastable β-Ti alloy mainly include EBW and LBW [13]. In addition, both solid-state phase transformation and microstructure evolution of Ti alloy show complexity and diversity, and thus they are always of concern to scholars. Sabol et al. (2014) effectively explained the microstructure evolution of EBW welded joints of metastable β-Ti alloy (Ti5553) and its influence on fracture behavior of the joints [14,15,16]. Shi et al. (2007) explored the fracture toughness of LBW joints of metastable β-Ti alloy (Ti-6.5Al-2Zr-1Mo-1V) [17]. Shariff et al. (2012) investigated evolution of the microstructure and performance of the welded joints of metastable β-Ti alloy (Ti5553) in wire filling laser welding and suggested that the wire filling LBW method caused quality improvement of welding joints of metastable β-Ti alloy [18].

Due to its advantages, including high energy density, small heating zone and insignificant welding deformation, LBW is extremely applicable for welding metastable β-Ti alloy [19]. Sánchez-Amaya et al. (2017) conducted Ti-5Al-5V-5Mo-3Cr butt joints through LBW within a conduction regime. The results indicated that the obtained LBW joint showed higher hardness of the FZ and higher values of ultimate tensile strength than those joints previously obtained with other joining processes [20]. However, in-depth investigations on the change of microstructures in the FZ during the LBW process need to be clarified. Moreover, the relationship between PWHT and the performance of LBW joints remains to be identified. In this study, the influences of welding parameters and PWHT on microstructures and the performance of laser-welded joints of metastable β-Ti alloy (Ti55531) were explored to provide guidance on the application of Ti55531 alloy in welded structures. 

## 2. Materials and Methods

**Figure 1:**
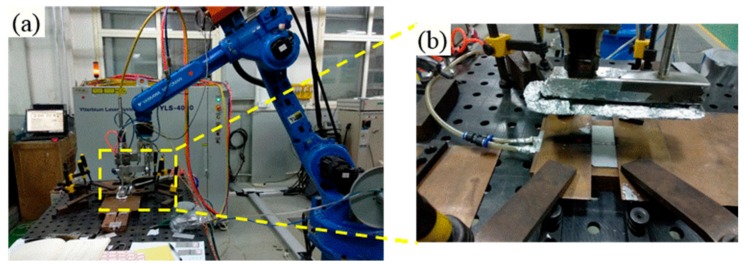
(**a**) Robot-based laser welding system, and (**b**) clamping device.

A sheet with the dimensions of 90 mm × 40 mm × 2 mm was cut from the forge piece of Ti alloy—Ti55531 (Ti-5Al-5Mo05V-3Cr-1Zr) and processed through solid solution and aging. Before the welding, the sheet was immersed into pickling liquor (10 mL HNO_3_, 20 mL HF, and 50 mL H_2_O), to remove the surface oxide film, and then washed in acetone. The welding experiments were carried out by utilizing an IPG YLS-4000 fiber laser system (Apache, San Francisco, CA, USA), with a focal spot diameter of 0.2 mm and a focal length of 150 mm. During welding, argon was served as shielding gas at a constant flow rate of 30 L/min. The tilting angle of the welding head regarding the surface of the workpiece was 5°, as shown in Figure 1. The PWHT was conducted by applying a TCGC-1200 vacuum-tube furnace (CHEM, Shenzhen, China). During the PWHT, argon was injected in order to prevent workpieces from being oxidized. In order to optimize the parameters of LBW, a three-factor and five-level orthogonal experiment scheme was designed, taking power, welding speed and defocusing amount as variables, as shown in Table 1. The weld penetration, bead appearance and tensile strength were set as optimized responses in the statistical analysis: only when a full penetration joint without obvious weld defect (such as weld leakage) was formed was the tensile strength measured. The as-welded joint with the optimal mechanical performance was selected for PWHT. The parameters of PWHT were as follows: the sample was held for one hour at 830 °C at first, then cooled down to 550 °C in air and finally held for 7 h at 550 °C.

**Figure 2:**
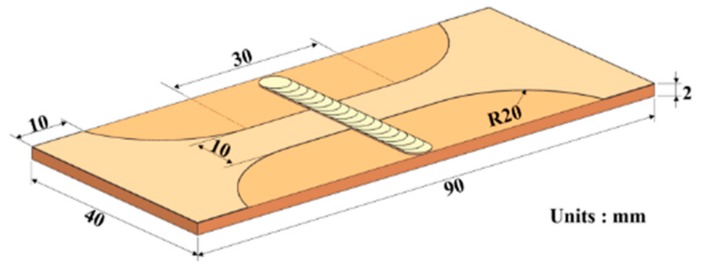
Dimensions of the sample for tensile test.

By using the standard procedures, the cross-sectional metallographic specimen of the welded joint was prepared and then etched with an etchant made of 8 mL HNO_3_, 2 mL HF, and 90 mL H_2_O. By using a Nikon MA200 optical microscope (Tokyo, Japan) and a LS-JLLH-22 scanning electron microscope (SEM, JEOL, Tokyo, Japan), the microstructures of cross-section of welded joint were observed and the precipitates of different zones in the welded joint were investigated by employing a TEM6000 transmission electron microscope (TEM, JEOL, Tokyo, Japan). By utilizing a semi-automatic microhardness tester (Qness, Melbourne, Austria), the microhardness distribution on cross-sections of welded joints were measured under a load of 200 gf and a dwelling time of 15 s. By using a CSS-88100 universal machinery testing machine (SINOMACH, Beijing, China), the tensile test was carried out on the welded joint at a tensile speed of 1 mm/min. The dimension of the sample of the welded joint used for tensile tests is shown in Figure 2. Moreover, the morphologies of tensile fracture of the sample was observed under the LS-JLLH-22 SEM.

## 3. Results and Discussion

### 3.1. Optimization of Welding Parameters

**Figure 3:**
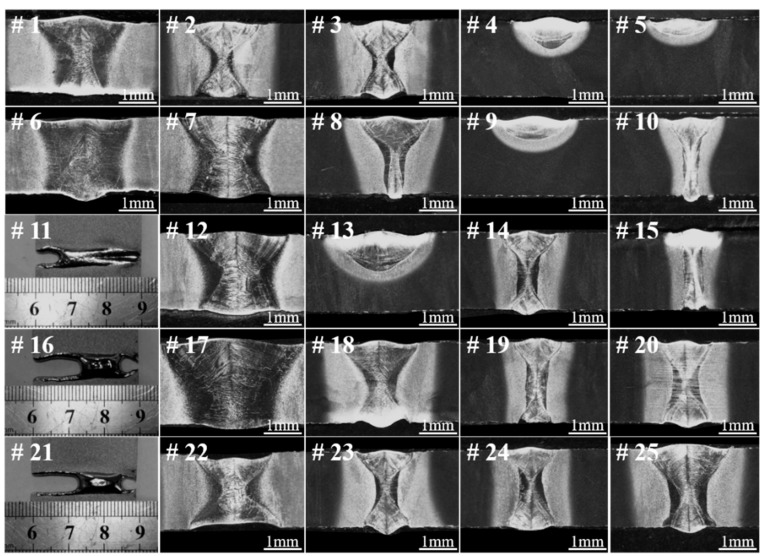
Morphologies of cross-sections of welded joints obtained by conducting welding experiments according to Table 1.

Table 1 shows the orthogonal experiment scheme for LBW of Ti55531 alloy and tensile strengths of welded joints achieved under various welding conditions. Figure 3 displays the morphologies of cross-sections of the welded joints obtained by conducting the LBW method according to Table 1. By carrying out range analysis and variance analysis on results obtained through orthogonal experiment, it can be seen that the defocusing amount (*f*) most significantly influences the tensile strength of the welded joint, followed by welding speed (*V*), and laser power (*P*) shows the least influence under the conditions considered in the study.

In Table 2 (i.e., range analyzed results), the *k_i_* indicated the sum of tensile strength among the joints conducted under *i* level of a factor. Then the range (*R*) was obtained by calculation of the difference value between the maximum and minimum of *k_i_.* The larger the *R*, the greater the influence of a parameter on tensile strength. From Table 2, it can be seen that defocusing amount (*f*) most significantly influenced the tensile strength of the welded joint, followed by welding speed (*V*), and laser power (*P*). The results of analysis of variance (ANOVA) are listed in Table 3. Then F_0.05_ (4, 4) = 6.39 was used to detect the significance of each factor to the responses. It was found that all the F-value of each factor were smaller than F_0.05_ (4, 4), which indicated that the influence of *f, V* and *P* on tensile strength was not significant.

**Figure 4:**
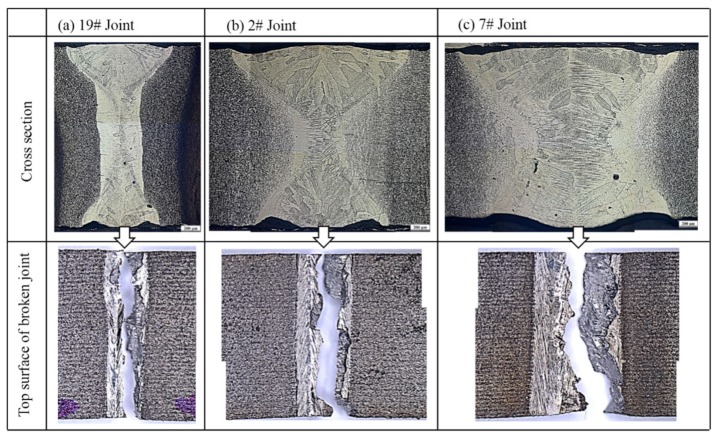
Morphologies of cross-sections of typical as-welded joints under OM. (**a**) #19: *P* = 3.5 kw, *v* = 4 m/min, *f* = 0 mm; (**b**) #2: *P* = 2 kw, *v* = 2 m/min, *f* = 0 mm, and (**c**) #7: *P* = 2.5 kw, *v* = 2 m/min, *f* = +3 mm.

Figure 4 shows the morphologies of cross-sections before fracture and top surfaces after fracture of three typical as-welded joints with great difference in morphologies of FZ and tensile strengths larger than 800 MPa. It can be seen from the figure that the shapes of the FZs of three welded joints all showed wide top and bottom parts while a narrow middle part. More specifically, it can be seen that the top part was slightly wider than the bottom, similar to an hourglass shape, which was also reported by Shariff [21]. The shape of keyhole (i.e., blind keyhole or through keyhole) has a great influence on the weld morphology. Zhang et al. has demonstrated that the transient behavior of the keyhole has an obvious cyclical characteristic during laser full penetration welding, which could result in that the top part of the weld seam being wider than the bottom [22]. According to the test result of tensile strength in Table 1, it can be inferred that the tensile strength of the welded joint declined with the growth of width of the FZ. As shown in Figure 4, the FZs of the three types of joint all consisted of columnar crystal microstructures and the wider the FZ, the larger the size of the columnar crystals [20]. Previous researches showed that the size gradient of microstructures of laser-welded joints had a significant influence on the performance of the joints [23,24]. Generally, the location where the size of microstructures shows the most significant change is the weakest part of a welded joint. As shown in Figure 4, the locations with the most dramatic change of grain size in the three types of welded joints were all found at the interface between the FZ and HAZ. Moreover, the wider the FZ, the larger the size gradient of microstructures at the interface between the FZ and HAZ. By observing the surface morphologies of the fractured welded joints in Figure 4, it can be seen that the three types of laser-welded joints at as-welded condition were all fractured at the vicinity of the interface between the FZ and HAZ.

### 3.2. SEM Observation on Microstructure of Laser-Welded Ti55531 Joints

#### 3.2.1. As-Welded Ti55531 Joint

**Figure 5:**
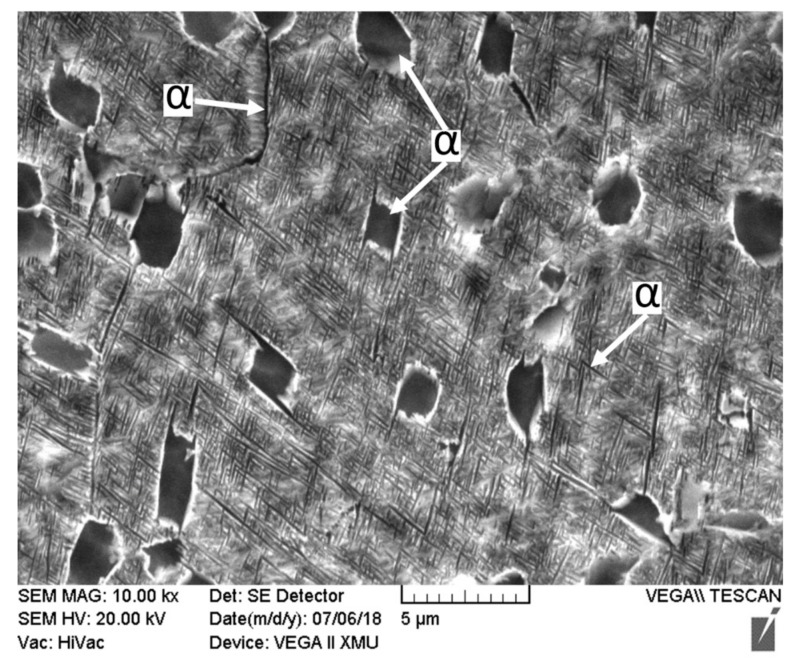
Microstructure of base metal of Ti55531 alloy.

**Figure 6:**
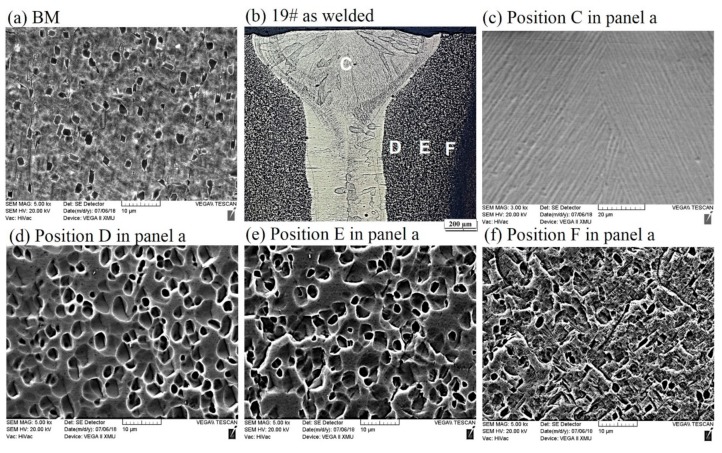
Microstructures of (**a**) BM, and (**b**) cross sections of as-welded Ti55531 joints, accompany with high magnification figures (**c**–**f**).

Figure 5 shows the microstructures of Ti55531 BM and Figure 6 displays the microstructures of various zones of laser-welded Ti55531 joints. It can be seen from Figure 5 that three kinds of α phase were observed in BM, which included equiaxial primary α_p_ phase, lamellar secondary α_S_ phase precipitated from β-phase matrix, and α_GB_ phase at grain boundaries [11]. Moreover, the volume fraction of α_p_ was about 6%~7%, and with 2~3 μm in size.

By taking 19# (of which the tensile strength is 940 MPa) welded joint as an example, the influence of welding process on microstructures of Ti55531 alloy was analyzed. As shown in Figure 6c, owing to the high temperature of molten pool, α phases were completely dissolved during the welding process. During the rapid cooling process of the FZ, β phase failed to transform into α phase while transformed into supersaturated solid solution with the same component while different crystal structures as parent phase. When the FZ was cooled to room temperature after welding, 100% of β phases remained in the FZ. It had been validated that various metastable-β alloys (i.e., Ti-15V-3-Cr-3Al-3Sn, Ti-8V-7Cr-3Al-1Zr and Ti-8V-4Cr-2Mo-2Zn) conformed to the phenomenon. 

As shown in Figure 6b, owing to the temperature of the HAZ rising, lamellar α_S_ phase and α_GB_ phases in the HAZ (i.e., position D and E in Figure 6a) close to the FZ completely disappeared. Only granular microstructures with the size of about 3–5 μm were found in the HAZ. Through analysis, it can be speculated that the granular microstructures were formed after original equiaxial α phases were heated and then partially dissolved. By comparing Figure 6d–f, it can be seen that the closer to the FZ, the larger the size of granular microstructures in the HAZ and the fewer the lamellar α_S_ phases. It is worth noting that during LBW, the HAZ underwent a weld thermal cycle: rapidly heating and rapidly cooling. The peak temperature of the HAZ during the weld thermal cycle was lower than the liquid temperature of the BM while probably higher than the transition temperature of β phase. Therefore, α phase in the HAZ was dissolved. Additionally, the locations in the HAZ with different distances from the center of the welded seam showed different peak temperatures and the closer to the FZ, the higher the peak temperature of the location. Therefore, the closer to the FZ, the more sufficiently the α phase dissolved. 

**Figure 7:**
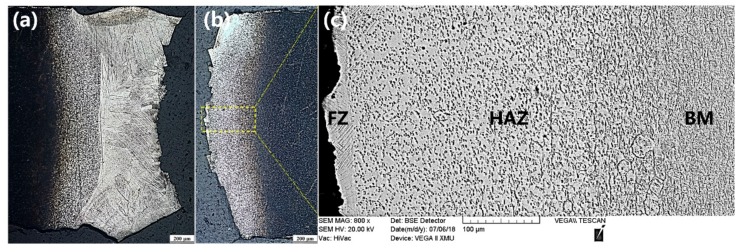
Fractured location of as-welded 19# joint (**a**,**b**) and corresponding macrostructure displayed in (**c**).

Figure 7 shows the morphology of the cross-section of 19# as-welded joint after undergoing tensile fracture. It can be seen that the sample was fractured at one side of the FZ in the vicinity of the interface between the FZ and the HAZ. According to the figure, it can also be clearly seen that the closer to the FZ, the more sufficiently the α phase in the HAZ dissolved. 

#### 3.2.2. Ti55531 Joint after PWHT

**Figure 8:**
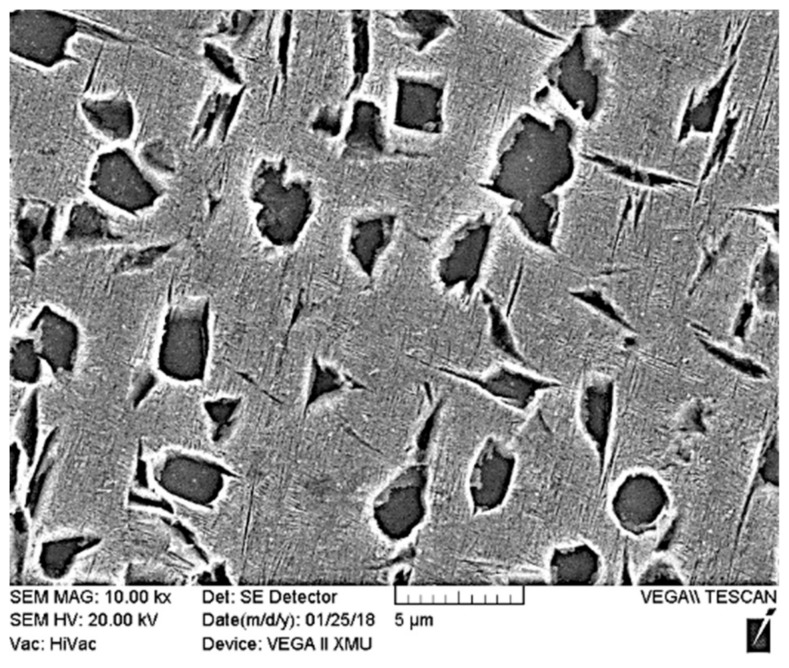
Microstructures of Ti55531 base metal (BM) after PWHT.

**Figure 9:**
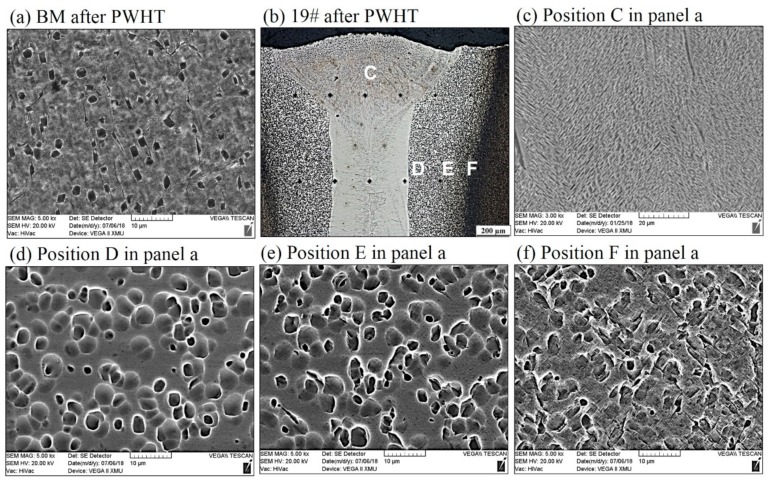
Microstructures of (**a**) BM after PWHT, and (**b**) cross sections of as-welded Ti55531 joint after PWHT, accompany with high magnification figures (**c**–**f**).

By taking 19# welded joint as an example, the influence of PWHT on the microstructures of a laser-welded Ti55531 joint was analyzed. Figure 8 shows the microstructures of BM of the Ti55531 joint after PWHT and Figure 9 displays those of various zones of laser-welded Ti55531 joints after PWHT. As shown in Figure 8, the three kinds of α phases distributed in the original BM were all significantly changed. After conducting PWHT, the number of equiaxial α_p_ phase distributed in β phase matrix increased and the interface between the equiaxial α_p_ phase and β phase matrix became blurred. Moreover, the size of α_p_ phase also significantly rose, to about 3–5 μm. Additionally, the lamellar secondary α_S_ phase precipitated from β phase matrix nearly totally disappeared and no continuous α_GB_ phases were found at the grain boundary. It can be inferred that the performance of BM would be weakened after PWHT.

Figure 9c shows microstructures in the FZ of the laser-welded Ti55531 joint after PWHT. It can be seen from the Figure that after undergoing PWHT, compact lamellar α phases re-occurred in the FZ. When conducting PWHT on the welded joint, the lamellar α phase was precipitated from β phase matrix and appeared as a stagger arrangement. It can be speculated that the mechanical performance of the FZ in laser-welded Ti55531 joints after PWHT would be improved.

Figure 9d–f displayed the microstructures of different positions in the HAZ of laser-welded Ti55531 joints after PWHT. As shown in the figure, the interface between granular phases seeming like to be α_p_ and β phase matrix in the HAZ became more blurred, with a smoother transition. Additionally, compared with as-welded joint, the size of granular phases in the HAZ increased after PWHT.

### 3.3. TEM Observation on Microstructure of Ti55531 Joint

**Figure 10:**
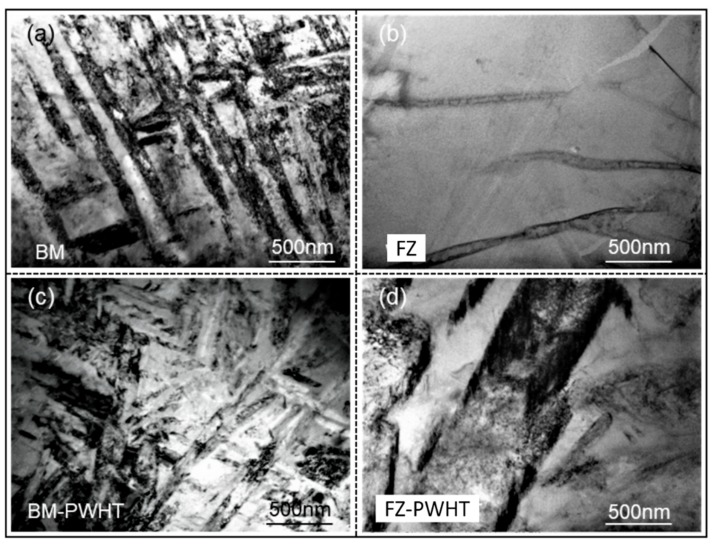
TEM observation on the precipitates of (**a**) BM, (**b**) FZ of as-welded Ti55531 joint, (**c**) BM after PWHT and (**d**) FZ of as-welded Ti55531 joint after PWHT.

Figure 10 shows the distributions of precipitates in as-welded joints and the joint being subjected to PWHT observed through TEM. It can be seen that the precipitated phase of as-welded BM in Figure 10a mainly appeared as lamellar α phase, with a great quantity. However, lamellar α phases in the FZ of the as-welded joint (Figure 10b) were hardly observed. The experimental results revealed that the hardness of the FZ was far lower than that of the BM. Moreover, as-welded joints showed the lowest overall tensile strength at 960 MPa and eventually were fractured at the FZ during tensile tests. After conducting PWHT (i.e., solution and aging process), part of lamellar α phases in the BM (Figure 10c) were dissolved after being subjected to PWHT, causing the mechanical performance of the BM to decline to some extent. New lamellar α phases re-occurred in the FZ (Figure 10d) of as-welded joints being subjected to PWHT, which certainly caused the strength of the FZ (Figure 10d) to strengthen after PWHT.

### 3.4. Mechanical Properties

#### 3.4.1. Microhardness

**Figure 11:**
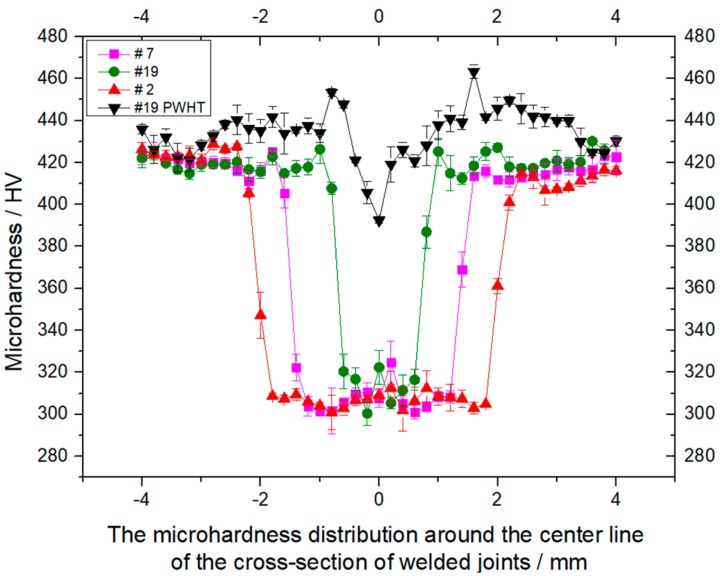
The influences of welding process and PWHT on the microhardness distribution of cross section of laser welded Ti55531 joints.

Figure 11 shows the influences of welding process and PWHT on the distribution of microhardness of cross-sections of laser-welded Ti55531 joint. It can be seen from the figure that PWHT has little influence on the microhardness of the BM: The microhardnesses of the BM before and after the PWHT were both about 420 HV. The microhardnesses of the FZs in three as-welded joints (i.e., 2#, 7# and 9#) had an insignificant difference, all about 300~320 MPa, implying that the welding process showed an insignificant influence on the microhardness of the FZ. In Figure 11, the widths of softening zones in three as-welded joints (i.e., 2#, 7# and 9#) were greatly different, mainly because the widths of the FZs under different welding parameters had a significant difference. The FZ of welded Ti55531 alloy was softened, which was mainly because all α phases in the FZ disappeared after the welding condition. A great quantity of lamellar α phases was precipitated from β phase in the FZ after being subjected to PWHT. Therefore, the microhardness of the FZ in laser-welded Ti55531 joints being subjected to PWHT was significantly improved, as shown in Figure 11. It can be seen from the figure that the microhardness of the FZ in laser-welded Ti55531 joints being subjected to PWHT was in the range of 390–440 HV in which the lowest microhardness appeared in the vicinity of the center of welded seam. 

#### 3.4.2. Tensile Test

**Figure 12:**
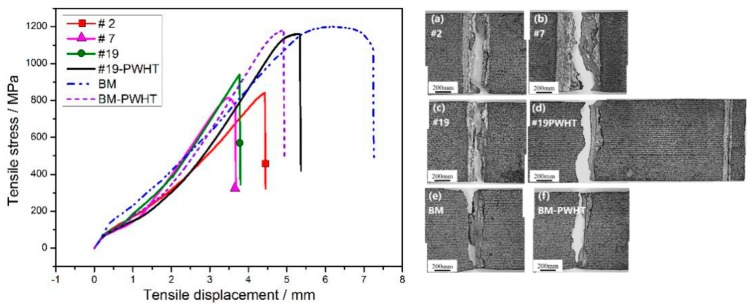
The stress–tensile displacement curve and fracture morphologies of Ti55531 laser welded. (**a**) #2: *P* = 2 kw, *v* = 2 m/min, *f* = 0 mm; (**b**) #7: *P* = 2.5 kw, *v* = 2 m/min, *f* = +3 mm; (**c**) #19: *P* = 3.5 kw, *v* = 4 m/min, *f* = 0 mm; (**d**) #19 after PWHT, (**e**) BM and (**f**) BM after PWHT.

Figure 12 shows the influences of welding process and PWHT on the tensile strength and tensile displacement before the fracture of laser-welded Ti55531 joints. At first, under the experimental conditions considered in this study, the tensile strength of the BM after undergoing PWHT was insignificantly changed while the tensile displacement before the fracture of the BM significantly declined, with a reduction amplitude of about 31.5%. Additionally, the tensile strengths and before-fracture tensile displacements of the three as-welded joints were all low. By taking the 19# welded joint as an example, the tensile strength and before-fracture tensile displacement of the joint separately took up 78.4% and 52.1% of those of the BM without being subjected to PWHT. After conducting PWHT, the tensile strength and before-fracture tensile displacement of the laser-welded Ti55531 joint both significantly increased, which separately accounted for 96.8% and 72.6% of those of the BM without being subjected to PWHT. As shown in Figure 12, the 19# laser-welded Ti55531 joint after the PWHT was fractured in the BM relatively far away from the welded seam during the tensile test, while the19# as-welded joint was fractured in the FZ during the tensile test. After conducting PWHT, part of the precipitated phases from the BM was dissolved, resulting in the slight reduction of the performance of the BM after the PWHT. However, lamellar α phases were re-precipitated from the FZ in the welded joint being subjected to PWHT, which caused the strengthening of the FZ after PWHT. Therefore, although 19# laser-welded Ti55531 joint was fractured in the BM relatively far away from the welded seam during the tensile test, the mechanical performance of the joint was still slightly lower than those of the BM without being subjected to PWHT.

#### 3.4.3. Fracture Morphology

**Figure 13:**
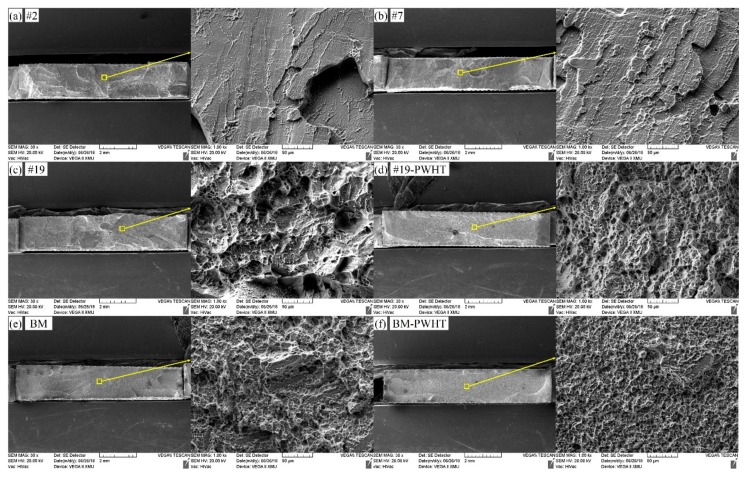
The tensile fractures of laser welded Ti55531 joints of (**a**) #2: *P* = 2 kw, *v* = 2 m/min, *f* = 0 mm; (**b**) #7: *P* = 2.5 kw, *v* = 2 m/min, *f* = +3 mm; (**c**) #19: *P* = 3.5 kw, *v* = 4 m/min, *f* = 0 mm; (**d**) #19 after PWHT, (e) BM and (f) BM after PWHT.

Figure 13 displays the fracture morphologies of six tensile samples mentioned in Figure 12 by using the SEM. It can be seen that the fractures of 2# and 7# joints belong to brittle fractures with smooth surfaces, showing typical cleavage step morphology. The tensile fracture of the as-welded 19# joint show an uneven surface while a few small dimples were presented. By contrast, the surfaces of fractures of three joints (i.e., 19#-PWHT, BM and BM-PWHT) were all uneven and dimples with different sizes and depths were all shown on the fractures, belonging to ductile fractures. Additionally, by comparing the fracture morphologies of three as-welded joints (the 2#, 7# and 9#), it can be seen that with increasing weld width, the smoother the surface of fractures of as-welded joints was, and the larger the tendency of brittle fracture. As a result, it can be inferred that welding condition and PWHT both significantly influence the mechanical performance of laser-welded Ti55531 joints [8]. 

## 4. Conclusions 

The following are the major conclusions that can be drawn from this study:

- Three kinds of α phases were observed in the BM of Ti55531 alloy: equiaxial primary α_p_ phase, lamellar secondary α_S_ phase precipitated from β-phase matrix, and α_GB_ phase at grain boundaries. The boundary line between α and β phases was clear. After laser welding, β phase in the FZ failed to be transformed into α phase and appeared in the whole of the FZ of the as-welded joint. The lamellar secondary α_S_ phases and α_GB_ phases at grain boundary in the HAZ of as-welded joints were nearly completely dissolved. The edge of equiaxial α_p_ phase in the HAZ of as-welded joint was significantly dissolved and the closer to the FZ, the more sufficiently the α phases in the HAZ were dissolved.
- After PWHT: The number of equiaxial α_p_ phases in β phase matrix of the BM increased and the interface between equiaxial α_p_ phases and β phase matrix became blurred, the size of α_p_ phases in BM significantly rose, and the lamellar secondary α_S_ phases and α_GB_ phase at grain boundary in β phase matrix nearly totally disappeared. After carrying out the PWHT, compact lamellar α phases re-occurred in the FZ and the interface between granular phases and β phase matrix in the HAZ became more blurred. Moreover, the size of granular phases in the HAZ increased.
- Welding condition had a significant influence on microstructures and the performance of laser-welded Ti55531 joints. Under a large weld heat input, the surface of tensile fracture of the welded joint was even, with typical cleavage step morphology. Under a low weld heat input, the tensile fractures of as-welded joints and joints being subjected to PWHT exhibited uneven morphology.
- Under the experimental conditions considered in the study, the microhardness of the BM was about 420 HV. The microhardness of the FZ in as-welded joints was in the range of 300~320 HV and increased to 390~440 HV after carrying out PWHT. Moreover, the tensile strength of as-welded joints was about 940 MPa, which took up 78.41% of that of the BM, while accounting for 96.8% after conducting PWHT.

## Figures and Tables

**Table 1:** Three-factor and five-level orthogonal experiment for continuous LBW of Ti55531.

Number	Laser Power *P* (kW)	Welding Speed *V* (m/min)	Defocusing Amount *f* (mm)	Tensile Strength (MPa)
1	2	1	−3	692.89
2	2	2	0	841.75
3	2	3	3	782.06
4	2	4	6	Lack of penetration
5	2	5	9	Lack of penetration
6	2.5	1	0	784.13
7	2.5	2	3	814.97
8	2.5	3	6	803.31
9	2.5	4	9	Lack of penetration
10	2.5	5	−3	725.26
11	3	1	3	Weld leakage
12	3	2	6	790.12
13	3	3	9	Lack of penetration
14	3	4	−3	808.27
15	3	5	0	856.10
16	3.5	1	6	Weld leakage
17	3.5	2	9	741.44
18	3.5	3	−3	817.92
19	3.5	4	0	940.52
20	3.5	5	3	703.78
21	4	1	9	Weld leakage
22	4	2	−3	843.90
23	4	3	0	720.41
24	4	4	3	853.88
25	4	5	6	880.09

**Table 2:** Range analyzed results of orthogonal experiment.

Range	Laser Power *P*	Welding Speed *V*	Defocusing Amount *f*
*K*_1_	463.32	295.39	777.65
*K*_2_	625.53	806.43	828.56
*K*_3_	346.90	624.74	630.93
*K*_4_	640.63	520.53	494.70
*K*_5_	659.66	632.21	148.29
*R* (range)	312.76	511.04	680.27

**Table 3:** Analysis of variance results of orthogonal experiment.

Factor	Degree of Freedom	*F*-Value	*p*-Value	Significance
Power	4	0.53	0.714	Not significant
Welding speed	4	2.23	0.126	Not significant
Defocusing amount	4	4.72	0.016	Not significant

## References

[1] Leyens C., Peters M. (2003). Titanium and Titanium Alloys: Fundamentals and Applications.

[2] Dikovits M., Poletti C., Warchomicka F. (2014). Deformation Mechanisms in the Near-β, Titanium Alloy Ti-55531. Metall. Mater. Trans. A.

[3] Yolton C.F., Froes F.H., Malone R.F. (1979). Alloying element effects in metastable beta titanium alloys. Metall. Mater. Trans. A.

[4] Warchomicka F., Poletti C., Stockinger M. (2011). Study of the hot deformation behaviour in Ti–5Al–5Mo–5V–3Cr–1Zr. Mater. Sci. Eng. A.

[5] Bykov V.A., Kulikova T.V., Vedmid’ L.B., Fishman A.Y., Shunyaev K.Y., Tarenkova N.Y. (2014). Thermophysical properties of Ti-5Al-5V-5Mo-3Cr-1Zr titanium alloy. Phys. Metals Metall..

[6] Barriobero-Vila P., Requena G., Schwarz S., Warchomicka F., Buslaps T. (2015). Influence of phase transformation kinetics on the formation of α in a β-quenched Ti–5Al–5Mo–5V–3Cr–1Zr alloy. Acta Mater..

[7] Guimarães R.P.M., Oliveira V.B., Barriobero-Vila P., Requena G., Pinto H.C. Preliminary studies on the aging kinetics of Ti-55531 alloy using high-energy synchrotron X-ray diffraction. Proceedings of the SICEM 2016.

[8] Huang C., Zhao Y., Xin S., Zhou W., Li Q., Zeng W. (2017). High cycle fatigue behavior of Ti–5Al–5Mo–5V–3Cr–1Zr titanium alloy with bimodal microstructure. J. Alloys Compd..

[9] Huang C., Zhao Y., Xin S., Zhou W., Li Q., Zeng W. (2017). Effect of microstructure on tensile properties of Ti–5Al–5Mo–5V–3Cr–1Zr alloy. Mater. Sci. Eng. A.

[10] Huang C., Zhao Y., Xin S., Tan C., Zhou W., Li Q., Zeng W. (2017). Effect of microstructure on high cycle fatigue behavior of Ti–5Al–5Mo–5V–3Cr–1Zr titanium alloy. Int. J. Fatigue.

[11] Boyer R.R. (1996). An overview on the use of titanium in the aerospace industry. Mater. Sci. Eng. A.

[12] Becker D.W., Baeslack W.A. (1980). Property-microstructure relationships in metastable-beta titanium alloy weldments. Weld. J. Res. Suppl..

[13] Pasang T., Amaya J.M.S., Tao Y. (2013). Comparison of Ti–5Al–5V–5Mo–3Cr Welds Performed by Laser Beam, Electron Beam and Gas Tungsten Arc Welding. Procedia Eng..

[14] Sabol J.C. (2014). Analysis of Microstructural Evolution and Fracture Mechanisms in Ti–5Al–5V–5Mo–3Cr–0.4Fe in Response to Electron Beam Welding and Post Weld Heat Treatments. Ph.D. Thesis.

[15] Sabol J.C., Pasang T., Misiolek W.Z. (2012). Localized tensile strain distribution and metallurgy of electron beam welded Ti–5Al–5V–5Mo–3Cr titanium alloys. J. Mater. Process. Technol..

[16] Marvel C.J., Sabol J.C., Pasang T., Watanabe M., Misiolek W.Z. (2017). Improving the Mechanical Properties of the Fusion Zone in Electron-Beam Welded Ti–5Al–5Mo–5V–3Cr Alloys. Metall. Mater. Trans. A.

[17] Shi Y., Fei Z., Li X., Gong S., Chen L. (2007). Effect of laser beam welding on fracture toughness of a Ti–6.5Al–2Zr–1Mo–1V alloy sheet. J. Mater. Sci..

[18] Shariff T., Chromik R.R., Wanjara P., Cuddy J., Birur A. (2012). Effect of joint gap on the quality of laser beam welded near-β Ti-5553 alloy with the addition of Ti–6Al–4V filler wire. J. Mater. Sci..

[19] Nunes A.C. (1985). A Comparison of the Physics of Gas Tungsten Arc Welding (GTAW), Electron Beam Welding (EBW), and Laser Beam Welding (LBW).

[20] Sánchez-Amaya J.M., Pasang T., Amaya-Vazquez M.R., Lopez-Castro J.D.D., Churiaque C., Tao Y., Pedemonte F.J.B. (2017). Microstructure and Mechanical Properties of Ti5553 Butt Welds Performed by LBW under Conduction Regime. Metals.

[21] Shariff T., Cao X., Chromik R.R., Baradari J.G., Wanjara P., Cuddy J., Birur A. (2011). Laser welding of Ti–5Al–5V–5Mo–3Cr. Can. Metall. Q..

[22] Zhang L.J., Zhang J.X., Gumenyuk A., Rethmeier M., Na S.J. (2014). Numerical simulation of full penetration laser welding of thick steel plate with high power high brightness laser. J. Mater. Process. Technol..

[23] Liu J., Gao X.L., Zhang L.J., Zhang J.X. (2014). A study of fatigue damage evolution on pulsed Nd:YAG Ti6Al4V laser welded joints. Eng. Fract. Mech..

[24] Shao C.D., Lu F.G., Cui H.C., Li Z.G. (2018). Characterization of high-gradient welded microstructure and its failure mode in fatigue test. Int. J. Fatigue.

